# Shoulder Muscle Strength and Neuromuscular Activation 2 Years after Reverse Shoulder Prosthesis—An Experimental Case Control Study

**DOI:** 10.3390/jcm9020365

**Published:** 2020-01-29

**Authors:** Anna Rienmüller, Nicola A. Maffiuletti, Hans-Kaspar Schwyzer, Andreas Eggspühler

**Affiliations:** 1 Department of Orthopedic and Trauma Surgery, Medical University Vienna, AT-1090 Vienna, Austria; 2 Human Performance Lab, Schulthess Clinic, CH-8008 Zurich, Switzerland; nicola.maffiuletti@kws.ch; 3 Department of Orthopedic Surgery, Schulthess Clinic, CH-8008 Zurich, Switzerland; hans-kaspar.schwyzer@kws.ch; 4 Department of Neurology, Schulthess Clinic, CH-8008 Zurich, Switzerland; andreas.eggspuehler@kws.ch

**Keywords:** reverse shoulder arthroplasty, neuromuscular activation, electromyography, muscle strength, deltoid muscle, humerus lengthening

## Abstract

Although reverse shoulder arthroplasty (RSA) has shown successful postoperative outcomes, little is known about compensatory activation patterns of remaining shoulder muscles following RSA. The purpose of this experimental case control series was to investigate shoulder muscle strength and neuromuscular activation of deltoid and teres minor muscles 2 years after RSA. Humerus lengthening, center-of-rotation medialization, maximal voluntary strength, and electromyographic (EMG) activity were compared between the operated and the non-operated side of 13 patients (mean age: 73 years). Shoulder muscle strength was significantly lower on the operated side for external rotation (−54%), internal rotation (−20%), and adduction (−13%). Agonist deltoid EMG activity was lower on the operated side for shoulder flexion, extension, and internal and external rotation (*p* < 0.05). Antagonist deltoid coactivation was higher on the operated side for external rotation (*p* < 0.001). Large correlation coefficients were observed between shoulder adductor strength asymmetry and both center-of-rotation medialization (r = −0.73) and humerus lengthening (r = 0.71). Shoulder abduction strength and neuromuscular activation were well preserved 2 years after RSA, while persistent strength and activation deficits were observed for shoulder adduction and internal and external rotation. Additional studies are required to elucidate shoulder neuromuscular activation patterns before and after RSA to support decision making for surgical, implant design, and rehabilitation choices.

## 1. Introduction

The concept of reversed shoulder arthroplasty (RSA) was introduced in the early 90s by Grammont and Baulot, with the intent of replacing the glenoid and the humeral joint surface and optimizing deltoid muscle function in the presence of rotator cuff tear arthropathy [1]. The central biomechanical principles of RSA are distalization of the deltoid insertion (deltoid lever arm lengthening or retensioning) and medialization of center-of-rotation (COR) of the glenohumeral joint, which would in turn compensate for the lack of rotator cuff [2,3].

Successful postoperative clinical outcomes have been reported after RSA for severe rotator cuff lesions associated with osteoarthritis or fracture sequelae [4]. Nonetheless, deltoid retensioning and COR medialization not only influence the biomechanical behavior of the shoulder [2] but they can also affect the remaining rotator cuff and deltoid muscles in terms of tension, force distribution, and force vectors within the muscles. In turn, this may chronically alter the neuromuscular function of the deltoid and of the remaining teres minor [5], potentially leading to increased fatty infiltration and reduced muscle strength. Together, these alterations can affect shoulder function and long-term clinical outcomes [6], despite a lack of evidence.

The purpose of this experimental case control series was to evaluate shoulder muscle strength and neuromuscular activation of deltoid and teres minor muscles 2 years after RSA. Quantitative surface electromyographic (EMG) activity and isometric shoulder torque associated to maximal voluntary contractions were therefore compared between the operated and the contralateral non-operated side in a series of patients who underwent primary RSA.

## 2. Materials and Methods

### 2.1. Subjects

The study protocol was approved by the local ethics committee (KEK-ZH-NR:2010-0225/0) and all procedures conformed to the Helsinki declaration. Patients who underwent primary RSA (Uncemented PROMOS® Reverse, Smith & Nephew, Rotkreuz, Switzerland) in our clinic between January and December 2008 were screened at the scheduled 2-year postoperative follow-up in 2010. The surgical technique for implantation of RSA followed a standardized protocol; all patients were operated on in a beach-chair position through a delto-pectoral approach. All components were implanted according to the manufacturer’s instructions [7] without cement and with the glenoid component implanted eccentrically. Post-operative rehabilitation followed an institutionally standardized treatment protocol consisting of two physical therapy sessions a week during a period of 3 months. In the first phase (from week 0 to week 2 post surgery), active and active-assisted mobilization of the operated arm limited to pain and visual field was allowed. The second phase (from week 3 to week 5 post surgery) consisted of active-resisted movements and active-dynamic stabilization. The third and last phase (from week 6 to week 12 post surgery) focused on coordination and strength in relation with activities of daily living.

Inclusion criteria were as follows: (1) primary RSA, (2) 20–30 months post-surgery, (3) age between 45 and 85 years, (4) “good” objective and subjective function of the operated shoulder allowing for pain-free (0 on a 0–10 visual analogue scale) movements within the range necessary to participate in the study (minimum active and passive flexion and abduction of 90°), (5) no previous muscle transfer surgery using latissimus dorsi or pectoralis major muscle on the operated side, (6) no radiological signs of loosening, scapular notching, or baseplate failures, (7) unaffected contralateral shoulder (minimum active flexion and abduction of 140° and pain free (visual analogue scale score = 0), ultrasonography negative for rotator cuff lesion and/or biceps tendon dislocation, not restricted during activities of daily living). In this way, the non-operated shoulder was treated as a healthy (control)-shoulder. Patients with cognitive (based on mini-mental-status-exam) and/or language deficits, as well as with contraindications for EMG activity and maximal strength assessment, were excluded. Out of 92 patients with complete follow-up data at 2 years, 13 met all inclusion criteria and were willing to participate in this study. Indications for surgery were rotator cuff tear arthropathy (i.e., a combination of glenohumeral osteoarthritis with a massive rotator cuff tear [8]) in 12 cases and trauma in one case. All subjects gave written informed consent prior to the investigation.

### 2.2. Range of Motion and Clinical Outcome Scores

Active range of motion in abduction, flexion, and internal and external rotation for both shoulders was measured at the 2-year follow-up (hereafter referred to as post-operative) by means of a plastic goniometer. The Constant score was evaluated both pre- and post-operatively by an orthopedic surgeon not involved in the study [9]. On the same occasions, patients were also asked to complete the self-reported QuickDASH questionnaire [10].

### 2.3. Radiographic Evaluation

**Figure 1:**
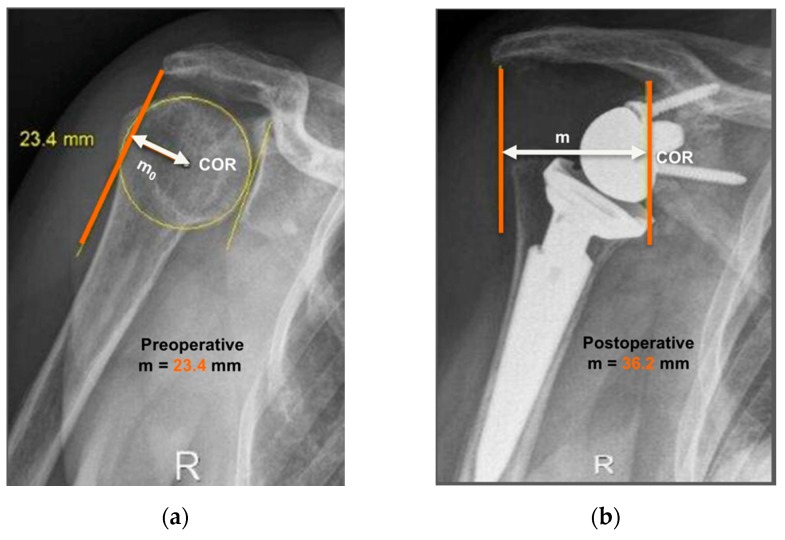
Anteroposterior radiographs of the same patient pre- and post-operatively are shown. Evaluation of center-of-rotation (COR) medialization was performed according to the protocol of Greiner et al. [5]. Pre-operatively (**a**), the distance “m_o_” was measured between the center of the circle involving the articular surface (COR) and the vertical line from the outer border of the acromion. Post-operatively (**b**), the distance “m” was measured between the center of the base plate (COR) and the vertical line from the outer border of the acromion. COR medialization was calculated as the difference between the distances “m_o_” and “m” as follows: ∆m = m_o_ − m. m_o_ > m was found in all subjects (12.8 mm for this representative patient).

**Figure 2:**
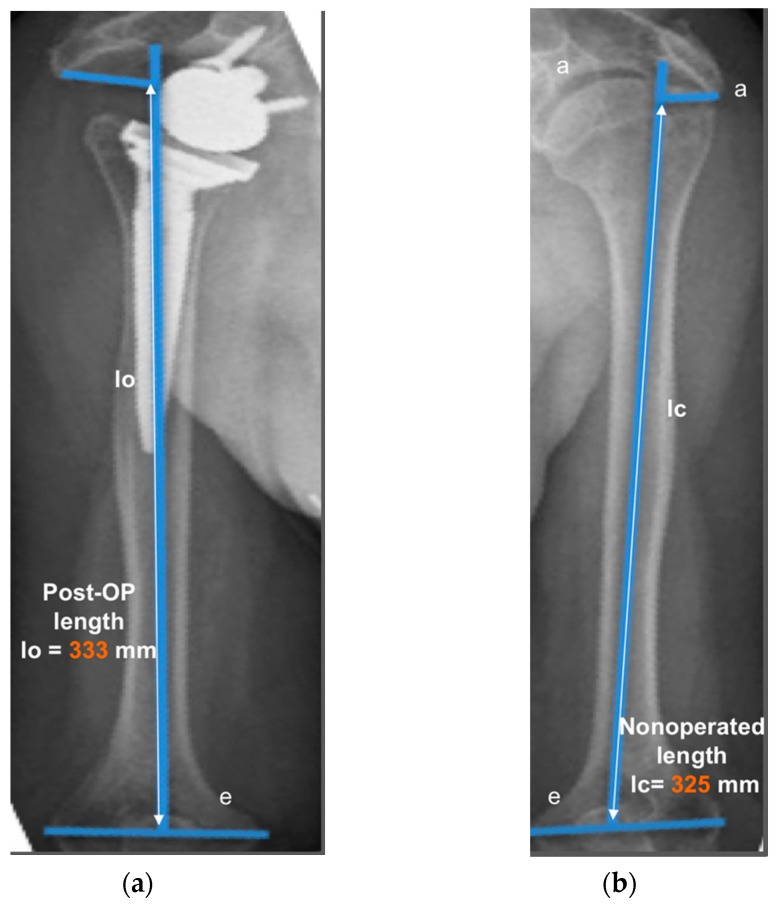
Measurement of humerus lengthening according to the protocol of Greiner et al. [5] and Lädermann et al. [10]. The humeral shaft axis was drawn, and the distance between the two lines “a” (subacromial line) and “e” (inter-epicondylar line) was measured as the humerus length for the operated (lo) (**a**) and non-operated side (lc) (**b**). The difference between the operated and non-operated humerus length (∆l = lo − lc) was considered as humerus lengthening if lo > lc. (8 mm for this representative patient).

The operated side was evaluated both pre- and post-operatively on true antero-posterior shoulder radiographs obtained in neutral rotation, according to the protocol described by Greiner et al. [5]. The COR was located in the center of the base plate and the distance “m” to a vertical line on the outer border of the acromion was measured (Figure 1). Humerus length was evaluated post-operatively for the operated and non-operated side (Figure 2) according to the protocol described by Lädermann et al. [11]. True antero-posterior radiographs were taken in neutral rotation of the arm with the patients standing. The distance between the midpoint of the epicondylar line and a perpendicular line passing through the shaft axis from the most lateral point of the acromion was measured. All measurements were blinded and performed twice by two trained orthopedic surgeons not included the operating surgeon. Consensus decision by mean was found a posteriori. The reliability of radiographic outcomes was moderate to good with intraclass correlation coefficients (intra-session and inter-observer) comprised between 0.74 and 0.86 [12].

### 2.4. Assessment of Muscle Strength

Hand/side dominance was documented before assessing muscle strength. Post-operative maximum voluntary strength of different shoulder muscles was evaluated separately for the operated and non-operated side using an isokinetic dynamometer (Biodex System 4 Pro, Biodex Medical Systems Inc., Shirley, NY, USA), which allowed recording of isometric torque in predefined positions. Subjects were placed in a sitting position and securely stabilized to the chair of the dynamometer using two crossover shoulder harnesses and one abdominal belt. The seat and lever arm of the dynamometer were adjusted individually according to the instructions of the manufacturers. Gravity correction was performed to account for the weight of the limb being tested. Three testing positions with two directions each were considered using the following settings:

- for abduction and adduction: starting position at 30° of abduction, neutral elbow position, neutral hand position, handle parallel to the chair;
- for flexion and extension: starting position at 30° of abduction, neutral elbow position, hand in 30° pronation;
- for internal and external rotation: starting position at 90° of abduction, elbow at 90° of flexion, hand in neutral position.

For all shoulder actions, participants received standardized verbal instructions and completed several familiarization trials at submaximal intensities (50–80% of their estimated maximal strength) on both sides. Then, participants performed three maximal voluntary contractions separated by 30 s, during which they were asked to contract their shoulder muscles as forcefully as possible with a gradual force build-up and relaxation within a total duration of 10 s. Visual feedback was provided to the participants as a real-time display of the isometric torque output. Testing positions and sides were randomly selected on an individual basis, and rest periods of approximately 3 min separated the test series conducted in the different positions and for the different sides. For each shoulder action, we quantified the peak torque from each of the three trials, as recorded by the Biodex software (sampling frequency: 100 Hz), and then averaged them.

### 2.5. Assessment of EMG Activity

**Figure 3:**
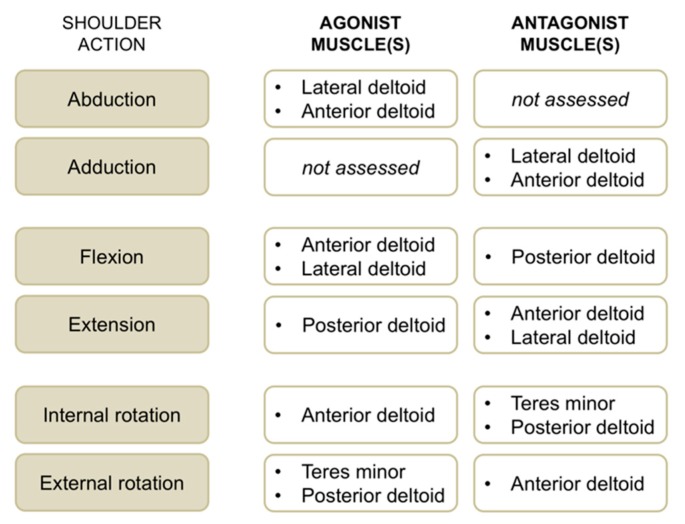
Overview of shoulder actions with respective agonist and antagonist muscles evaluated with surface electromyographic (EMG) activity in this study.

EMG activity of the anterior deltoid, lateral deltoid, posterior deltoid, and teres minor muscles was recorded concomitantly with maximum voluntary strength for the operated and non-operated side. We used single-differential bipolar surface electrodes (DE-2.1, Delsys Inc., Boston, MA, USA), with a distance between the recording stainless bars of 1 cm. Surface EMG sensors were positioned on respective muscle bellies according to SENIAM recommendations (http://www.seniam.org). A self-adhesive square (5 × 5 cm) ground electrode was placed at the wrist level. Self-adhesive tape was used to fix recording electrodes to the skin. Before the application of EMG sensors, the skin was shaved and cleaned with an alcohol preparation pad. All the selected muscles are superficial; hence, surface EMG activity is a good option for recording their activity [13]. EMG signals were amplified 1000 times and band-pass filtered (20 to 450 Hz) before being sampled at 1 kHz. EMG root mean square values were consistently calculated using a window length of 125 ms and a window overlap of 62.5 ms. For each contraction, the highest EMG activity obtained over a 1 s period (around peak torque) was calculated, and the average EMG activity of the three trials per condition was retained. For each muscle group and shoulder action (Figure 3), neuromuscular efficiency—that is the ratio between peak torque and EMG root mean square of agonist muscles [14]—was quantified to characterize agonist EMG activity. In a similar way, antagonist EMG activity was computed by normalizing submaximal EMG root mean square values for a given muscle when acting as an antagonist to the maximal EMG values of the same muscle when acting as an agonist (e.g., teres minor EMG activity recorded during internal rotation divided by teres minor EMG activity recorded during external rotation × 100). 

### 2.6. Statistics

Normal distribution of data was verified with Shapiro–Wilk tests. Paired t-tests (one-tailed) were used to examine (1) pre- to post-operative changes in clinical outcome scores and COR and (2) post-operative side-to-side (operated vs. non-operated) differences in range of motion, humerus length, muscle strength, and EMG activity. Pearson’s product-moment correlation coefficients (r) were calculated between radiographic variables (COR medialization and humerus lengthening) and side-to-side asymmetries in muscle strength. We considered correlation coefficients over 0.90 as nearly perfect, between 0.70 and 0.89 as very large, between 0.50 and 0.69 as large, between 0.30 and 0.49 as moderate [15]. Data are expressed as mean and SD (text and table) or SE (figures). The threshold for statistical significance was set to *p* < 0.05. Statistical analyses were performed with the SigmaPlot 11.0 software package (Systat Software Inc., Chicago, IL, USA).

## 3. Results

Patient demographics and clinical outcome scores are presented in Table 1. Constant and QuickDASH scores increased significantly post-operatively (*p* < 0.001). Post-operative shoulder abduction, flexion, and internal and external rotation ranges of motion were significantly lower for the operated than for the non-operated side (*p* < 0.001).

**Figure 4:**
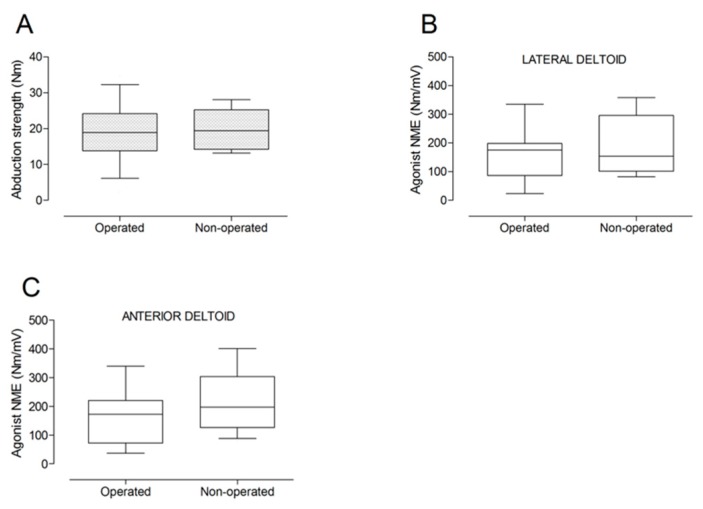
Box and whisker plots of shoulder abduction strength (**A**) and agonist neuromuscular efficiency (NME) of lateral deltoid (**B**) and anterior deltoid (**C**) muscles by side. The horizontal line in the box represents the median, the height of the box represents the interquartile range, and the distance between the opposite ends of the whisker represents the 10th–90th percentile.

**Figure 5:**
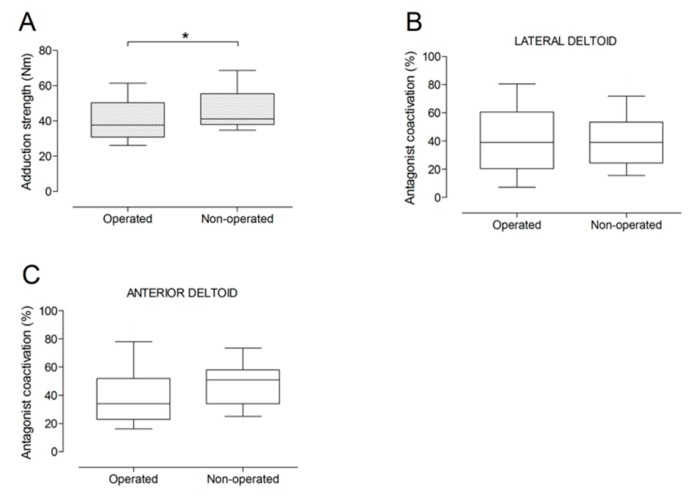
Box and whisker plots of shoulder adduction strength (**A**) and antagonist coactivation of lateral deltoid (**B**) and anterior deltoid (**C**) muscles by side. The horizontal line in the box represents the median, the height of the box represents the interquartile range, and the distance between the opposite ends of the whisker represents the 10th–90th percentile. *Operated < non-operated (*p* < 0.05).

**Figure 6:**
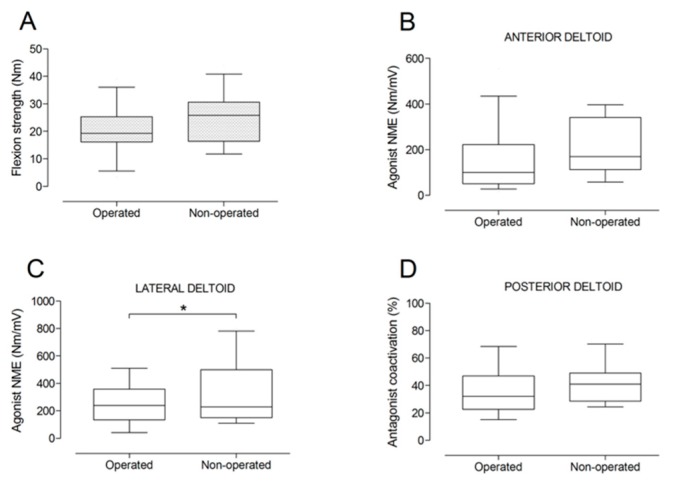
Box and whisker plots of shoulder flexion strength (**A**), agonist neuromuscular efficiency (NME) of anterior deltoid (**B**) and lateral deltoid (**C**) muscles, and antagonist coactivation of posterior deltoid muscle (**D**) by side. The horizontal line in the box represents the median, the height of the box represents the interquartile range, and the distance between the opposite ends of the whisker represents the 10th–90th percentile. *Operated < non-operated (*p* < 0.05).

**Figure 7:**
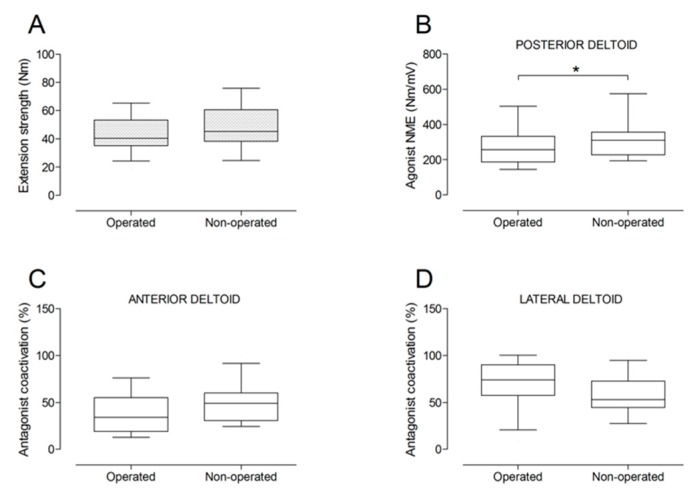
Box and whisker plots of shoulder extension strength (**A**), agonist neuromuscular efficiency (NME) of posterior deltoid muscle (**B**), antagonist coactivation of anterior deltoid (**C**) and lateral deltoid (**D**) muscles by side. The horizontal line in the box represents the median, the height of the box represents the interquartile range, and the distance between the opposite ends of the whisker represents the 10th–90th percentile. *Operated < non-operated (*p* < 0.05).

**Figure 8:**
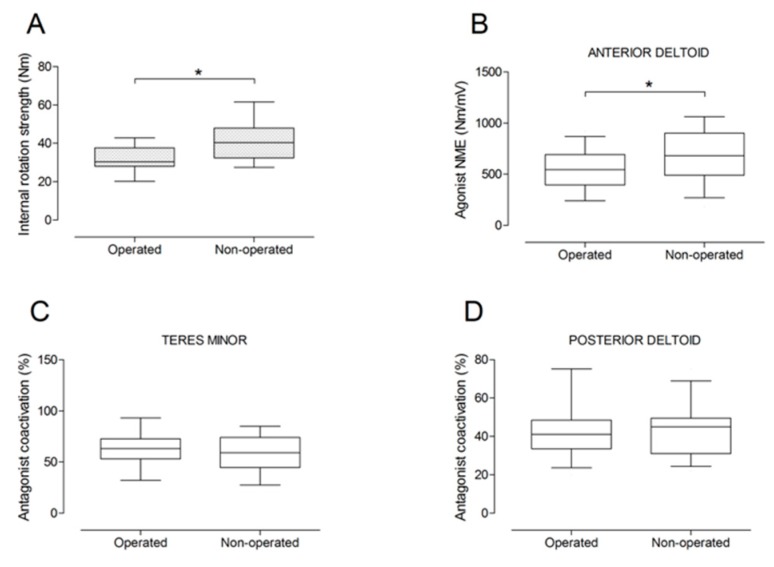
Box and whisker plots of shoulder internal rotation strength (**A**), agonist neuromuscular efficiency (NME) of anterior deltoid muscle (**B**), antagonist coactivation of teres minor (**C**) and posterior deltoid (**D**) muscles by side. The horizontal line in the box represents the median, the height of the box represents the interquartile range, and the distance between the opposite ends of the whisker represents the 10th–90th percentile. *Operated < non-operated (*p* < 0.05).

**Figure 9:**
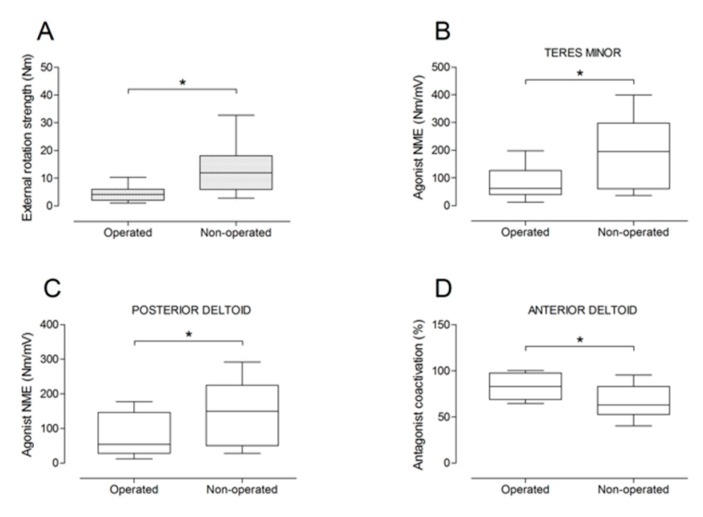
Box and whisker plots of shoulder external rotation strength (**A**), agonist neuromuscular efficiency (NME) of teres minor (**B**) and posterior deltoid (**C**) muscles, and antagonist coactivation of anterior deltoid muscle (**D**). The horizontal line in the box represents the median, the height of the box represents the interquartile range, and the distance between the opposite ends of the whisker represents the 10th–90th percentile. * Operated ≠ non-operated (*p* < 0.05).

There were no significant differences between the operated and the non-operated side for shoulder abduction strength and agonist EMG activity (Figure 4). Shoulder adduction strength was significantly lower on the operated side (−13%; *p* = 0.009), but no side-to-side differences were observed for antagonist EMG activity (Figure 5). No side-to-side differences were observed for shoulder flexion strength, agonist EMG activity of anterior deltoid, and antagonist EMG activity, while agonist EMG activity of lateral deltoid was significantly lower on the operated side (*p* = 0.026; Figure 6). Shoulder extension strength and antagonist EMG activity did not differ significantly between the two sides, while agonist EMG activity was significantly lower on the operated side (*p* = 0.037; Figure 7). For shoulder internal rotation, both muscle strength (−20%; *p* = 0.003) and agonist EMG activity (*p* = 0.025) were significantly lower on the operated side, while no side-to-side differences in antagonist EMG activity were observed (Figure 8). Shoulder external rotation strength (−54%; *p* < 0.001) and agonist EMG activity (*p* < 0.001) were significantly lower on the operated side, while antagonist EMG activity was significantly higher on the operated side (*p* < 0.001; Figure 9).

**Figure 10:**
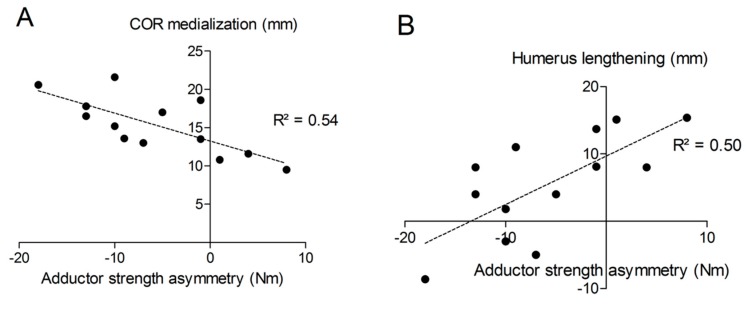
Correlations between shoulder adductor strength asymmetry and COR medialization (**A**) or humerus lengthening (**B**).

The distance “m” from COR consistently increased post-operatively (from 20.9 ± 3.3 mm to 36.2 ± 4.0 mm; *p* < 0.001) with a mean COR medialization of 15.3 ± 3.7 mm. Humerus length was longer on the operated (327 ± 21 mm) than on the non-operated side (321 ± 18 mm) with a mean humerus lengthening of 5.6 ± 7.7 mm (*p* = 0.011). No significant correlation was found between COR medialization and humerus lengthening (*p* > 0.05). Very large correlation coefficients were observed between shoulder adductor strength asymmetry and both COR medialization (r = −0.732; *p* < 0.001; Figure 10A) and humerus lengthening (r = 0.705; *p* < 0.001; Figure 10B). For all the other muscle groups, no significant correlations were observed between radiographic findings and strength asymmetries.

## 4. Discussion

To the best of our knowledge, this is the first experimental study evaluating shoulder muscle strength and neuromuscular activation following RSA with concomitant recordings of isometric joint torque and surface EMG activity during different shoulder actions realized on the three planes. Two years after RSA, we observed shoulder muscle weakness (i.e., reduced muscle strength) in adduction and internal and external rotation, whereas abduction, flexion, and extension strength were apparently unaffected. Agonist neuromuscular efficiency was significantly reduced for lateral deltoid (during shoulder flexion), posterior deltoid (during extension and external rotation), anterior deltoid (during internal rotation), and teres minor (during external rotation) muscles, while antagonist coactivation was increased for the anterior deltoid muscle during external rotation. Interestingly, shoulder adductor muscle weakness strongly correlated with both COR medialization and humerus lengthening.

Our current findings support the principle that RSA increases deltoid moment arms, thereby lowering the muscle force necessary to generate elevation torque [16] and allowing for regained abduction strength on the operated side. Similarly, Walker et al. found increased deltoid muscle activation after RSA, suggesting that this muscle is working “harder” post-operatively [17]. We also observed significantly higher raw EMG activity levels for the anterior deltoid (during shoulder abduction, flexion, and external rotation) and posterior deltoid (during extension and internal rotation) following RSA. Pegreffi et al. recorded deltoid EMG activity pre- and post-operatively and showed an absence of posterior deltoid activation during shoulder movement at the 2-year follow-up [18]. In contrast, Li et al. found good correlation between pre-operative posterior deltoid muscle activation and post-operative active range of motion in external rotation [19]. Although we found significantly less activation of the posterior deltoid muscle in external rotation and extension on the operated side, we cannot confirm the absence of activation, as we found symmetrical activation in internal rotation and flexion when comparing the operated to the contralateral shoulder.

In the current study, the most considerable alterations of both muscle strength and EMG activity variables were observed for external rotation. Interestingly, not only neuromuscular efficiency of the agonist muscles was considerably reduced for this action, but antagonist coactivation of the anterior deltoid muscle was also significantly increased, which further contributed to reducing external rotation torque. Shoulder muscle weakness in external rotation was therefore due to a combination of factors: (1) biomechanical changes due to COR medialization and humerus lengthening, (2) “dysfunction” of agonist muscles (posterior deltoid and teres minor), and (3) increased antagonist coactivation. Reverse shoulder arthroplasty was designed to increase lacking shoulder abduction without functional rotator cuff. More specifically, RSA was designed to increase abduction while at the same time reduce bending/compression forces at the glenosphere-bone interface [20]. However, medialization is associated with scapular notching, loss of shoulder contour, and reduced overall range of motion [21,22]. Deltoid moment arm is increased by 20–42% [21], and both anterior and posterior deltoid are recruited to also serve as abductors. Such recruitment of deltoid muscle fibers to initiate flexion and abduction comes, however, at the expense of axial rotation. Thus, the posterior deltoid may partially lose its function as an external rotator. Often, external and internal rotation remain unchanged or reduced after RSA, which can occur as a result of decreased moment arms of the subscapularis and teres minor muscles after RSA [23]. These muscles are further compromised by a decreased origin-to-insertion distance, resulting in less muscle tension. Interestingly, Valenti et al. [24] were able to show better internal and external rotation strength by less medializing the COR.

Several reasons could be responsible for the lower activation of the posterior deltoid muscle. On one hand, it may be due to a biomechanical reason in relation with the orientation of the scapula and glenoid with respect to the position of the body while performing external rotation and internal rotation at 90° of abduction. The humeral shaft is not oriented perpendicular to the surface of the glenoid; therefore, further contraction of the posterior deltoid muscle would lead to increased anterior shear forces causing subluxation of the humerus instead of creating external rotation. On the other hand, the design and implantation of the glenosphere and the orientation of the humeral shaft may play a role in affecting joint forces while performing external and internal rotation. Further studies are necessary to describe the role of each component while assessing posterior deltoid muscle function.

Coactivation of the anterior deltoid during external rotation in 90° of abduction might be due to necessary stabilization and centering of the shoulder joint after RSA. Shoulder dislocation after RSA is a common problem [25]. In 90° of abduction, external rotation force with activation of the posterior deltoid and posterior rotator cuff muscles as the main muscles of external rotation [26] will create anterior shear forces in the shoulder joint possibly leading to subluxation or dislocation of the shoulder. None of our patients reported shoulder dislocation following surgery nor discomfort during external rotation strength testing. These anterior shear forces might potentially be compensated by active shoulder stabilization using the anterior deltoid muscle. Further investigations are, however, necessary to analyze such compensatory stabilization patterns during different shoulder actions. In a recent computational modelling of sequential tear in rotator cuff muscles in RSA, Ackland et al. demonstrated superior shear forces during early abduction in the absence of rotator cuff muscles, despite relatively low antero-posterior and craniocaudal shear forces during abduction with a peak at 90° abduction, where joint compression favors over shear forces [27].

Correlational analyses showed that approximately 50% of the variance in shoulder adductor muscle weakness was explained by both COR medialization and humerus lengthening (R^2^ of approximately 0.5). This means the more medial the COR, the larger the strength asymmetry between the operated and non-operated shoulder; and the greater the lengthening of the humerus, the smaller the strength asymmetry. This was not the case, however, for the other shoulder movements tested. To the best of our knowledge, so far, no information is available on shoulder adduction strength following RSA. Biomechanically, it seems logical that a medialized COR would lead to better abduction strength at the expense of reduced adduction strength. The observed mismatch between the lack of EMG changes in the presence of reduced adduction strength suggests that muscles are approximately working the same and weakness is mainly due to biomechanical changes related to both COR medialization and humerus lengthening. So far, only a few studies evaluated biomechanical changes due to COR medialization and humerus/arm lengthening. Arm lengthening < 20 mm is usually recommended to avoid neurological impairments, while arm shortening and therefore inadequate tensioning of the deltoid may increase the risk of dislocation and poor anterior active elevation [28,29]. In our current study, humeral lengthening was comprised between -9 and +15 mm after RSA, which is consistent with the existing literature [11,28,29]. Positioning of the glenoid component is an important factor in relation to function and long-time survival of the implant [2,30]. All subjects tested in our study had eccentric glenospheres implanted. The eccentric design/positioning of the glenosphere has been shown to improve abduction and adduction range of motion and reduce the incidence of scapular notching [31].

Currently, there is no standardized pre-operative planning technique for appropriate implant position in relation to deltoid tension and humerus lengthening. Existing intra-operative criteria to assess shoulder stability are rather subjective [28]. Sufficient deltoid tension is required to dynamically stabilize the gleno-humeral joint, whereas excessive lengthening might lead to acromial fractures [32]. The deltoid muscle, which is the primary activator of the shoulder joint, not only plays an important role in functional movements but also in joint stability [9]. Adequate tensioning of this muscle is therefore directly related to its neuromuscular activation pattern and force generation capacity. Despite its critical role, however, little is known about the contribution of the deltoid muscle to shoulder function, also because normative EMG data of the different shoulder muscles are not available [16].

The present study has several limitations. No pre-operative strength and EMG assessments were conducted, and the comparison was restricted to the operated and non-operated shoulders of the same patients at a single follow-up interval. Although comparison of humeral length between the operated and contralateral side can be subject to possible bias, we still believe it is a valid option for measuring lengthening as preoperative length is not always easy to define (e.g., in case of trauma or cuff arthropathy in severe degeneration). Further, Lädermann et al. found no significant difference between the length of both arms and concluded that the contralateral side can be used with confidence for measuring humeral length after RSA [11]. Hand/side dominance may also affect, at least in part, neuromuscular parameters such as maximal voluntary strength. In our study group, however, 8 out of 13 subjects were operated on their dominant side, which resulted in a relatively minor effect of dominance on side-to-side strength comparisons. The sample size was quite small due to restrictive inclusion criteria, mainly with respect to the contralateral non-operated side. The principal indications in the pre-selected 92 patients with complete follow-up data at 2 years were rotator cuff tear arthropathy (68%), rheumatoid arthritis (20%), and posttraumatic (10%). The main reasons for non-inclusion of a large proportion of these patients were difficulties in the contralateral shoulder (55%), no pain-free range of motion of the operated shoulder (27%), and unwillingness to participate in the study (18%). In terms of sample size, we measured a mean adduction strength of ~40 Nm in a preliminary subset of 10 patients with RSA which, compared to data from healthy volunteers (~46 Nm) [33], revealed that a total number of 12 operated versus 12 non-operated shoulders would have been required to detect a significant side-to-side difference in shoulder adduction strength with an adequate statistical power.

Pectoralis major or latissimus dorsi muscle activity was not evaluated in this study because we were primarily interested in deltoid function as the main muscle undergoing compensatory changes after RSA. Both pectoralis major and latissimus dorsi create inferior shear forces [34], while the pectoralis major also creates anterior shear forces after RSA [16]; therefore, they are responsible for shoulder adduction. Their contribution to our current findings would have been of interest particularly in relation to the observed shoulder adduction weakness.

## 5. Conclusions

In conclusion, shoulder abduction strength and the corresponding neuromuscular activation were well preserved after RSA, while significant deficits in muscle strength and neuromuscular efficiency were observed in shoulder adduction and internal and external rotation. Shoulder adductor weakness was highly correlated with both COR medialization and humerus lengthening. Although these post-RSA results are promising, so far, optimum individual deltoid and soft-tissue tensioning are not yet quantifiable; thus, a certain experience from the surgical team is still required to allow for optimal muscular compensations after RSA. However, further studies are necessary to evaluate the neuromuscular activation pattern of the deltoid muscle and of the remaining shoulder stabilizing muscles following RSA to support decision making for surgical, implant design, and rehabilitation choices.

## Figures and Tables

**Table 1:** Patient characteristics.

Variable	Mean ± SD (range)	
N	13 (7 women)	
Age (yrs)	73 ± 12 (48–87)	
Body mass index (kg/m^2^)	28 ± 4 (18–35)	
Postoperative follow-up (months)	24 ± 1 (23–26)	
Operated side	11 right (8 dominant)	
Clinical outcome scores	Pre-operative	Post-operative
Constant score (0–100)	39 ± 11 (21–59)	76 ± 9 (50–84) *
QuickDASH score (0–100)	48 ± 17 (29–90)	82 ± 16 (50–97) *
Active range of motion	Operated side	Non-operated side
Abduction (°)	130 ± 18 (100–160)	160 ± 11 (140–170) *
Flexion (°)	140 ± 7 (120–145)	170 ± 11 (150–180) *
Internal rotation (°)	30 ± 13 (20–60)	50 ± 7 (40–60) *
External rotation (°)	60 ± 13 (40–80)	80 ± 10 (60–90) *

* significant difference between pre- and post-operative or operated and non-operated (*p* < 0.05).
